# Colonic intussusception masquerading as diverticulitis: a case report of a 31 year old diagnosed with colorectal carcinoma after multiple presentations to the ED for episodes of acute diverticulitis

**DOI:** 10.1093/jscr/rjac104

**Published:** 2022-03-30

**Authors:** Molly A McNamara, Maria Ceron y Ceron, Andrea L Merrill, Syed G Husain

**Affiliations:** The Ohio State University College of Medicine, Columbus, OH, USA; Department of Surgery, University of Florida, Jacksonville, FL, USA; Department of Surgery, Boston Medical Center, Boston University School of Medicine, Boston, MA, USA; Department of Surgery, Division of Colon and Rectal Surgery, The Ohio State University Wexner Medical Center and James Cancer Hospital, Columbus, OH, USA

## Abstract

Colonic intussusception is a rare occurrence in adults, with few reported cases in the literature. Patients often present with nonspecific and vague symptoms leading to diagnostic challenges and delayed treatment. Given the high incidence of underlying malignancy associated with cases of adult intussusception, it is important for clinicians to be able to recognize and coordinate appropriate follow-up. A 31-year-old female presented to the emergency department four times over a month with left lower quadrant abdominal pain. Multiple computed tomography scans showed inflammation and diverticulitis of the mid-descending colon along with a short segment of colonic intussusception. A colonoscopy was performed due to concern for malignancy. A partially obstructing mass was found in the descending colon that could not be traversed. Biopsies revealed necrosis and no evidence of malignancy. However, given high suspicion for malignancy, the patient underwent a laparoscopic left colectomy, which revealed a pT3N1b colon adenocarcinoma.

## INTRODUCTION

Intussusception of the bowel is defined as the telescoping of a proximal segment of the gastrointestinal tract within the lumen of an adjacent segment. Colonic intussusception is a rare occurrence in adults, accounting for only 1% of all bowel obstructions and 5% of all intussusceptions [1]. Although 90% of childhood intussusception is idiopathic, intussusception in adults is highly associated with a pathologic lead point. Up to half of reported cases of intussusception in adults are attributable to a malignant tumor [2].

Intermittent abdominal pain and vomiting although, nonspecific, can be an indicator of colonic intussusception. Abdominal computed tomography (CT) is the preferred diagnostic imaging modality with some studies demonstrating up to 78% accuracy of abdominal CT in diagnosing intussusception in patients [1]. Once the diagnosis off colonic intussusception has been established, underlying etiology should be investigated with a colonoscopy followed by surgical resection if a lead point is identified.

We present a case report of a young adult female who presented to the emergency department (ED) four times over the course of a month complaining of abdominal pain and change in bowel habits. Recurrent imaging was concerning for diverticulitis and intussusception that only mildly improved with antibiotics and dietary modification. Colonoscopy and biopsy eventually revealed a pT3N1b colon adenocarcinoma.

## CASE PRESENTATION

**Figure 1:**
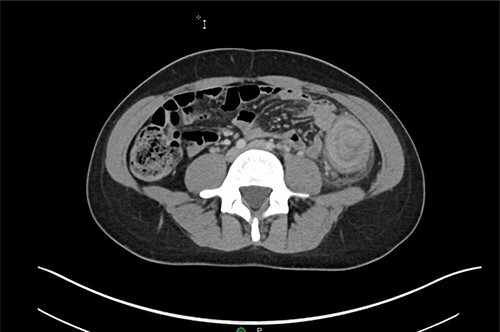
Axial CT demonstrating segmental wall thickening of the descending colon with adjacent inflammation favoring acute diverticulitis or segmental colitis.

**Figure 2:**
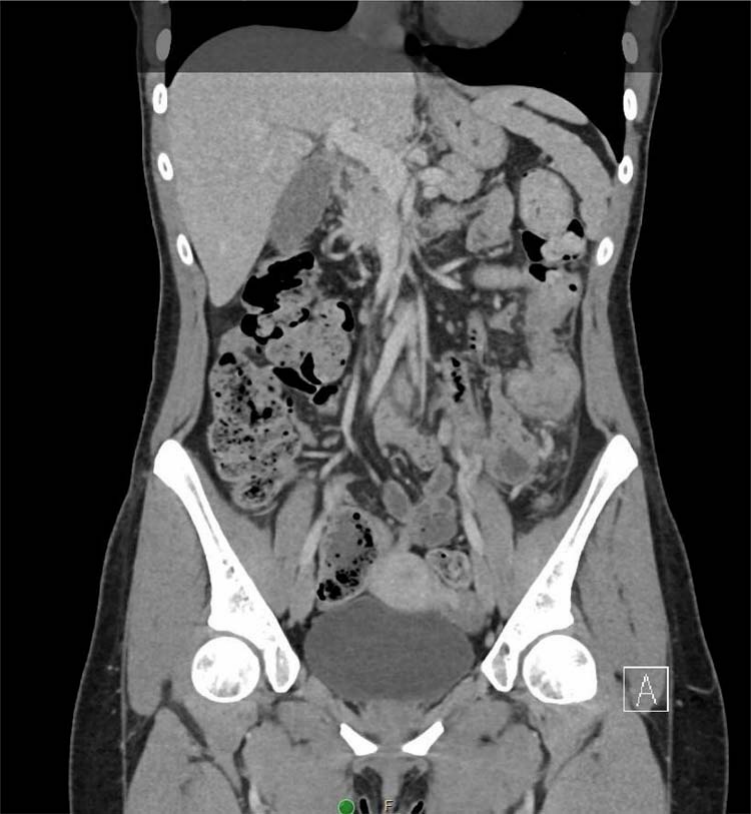
Coronal CT demonstrating segmental wall thickening of the descending colon with adjacent inflammation favoring acute diverticulitis or segmental colitis.

A 31-year-old woman initially presented to the ED with 2 days of left flank pain and a 1-year history of abdominal pain and bloating. The patient reported having had a fever (100.5 Tmax) and recurrent non-bloody loose stools. Vitals upon presentation to the ED were reassuring and the patient was afebrile. Initial physical exam was normal with the exception of costovertebral angle tenderness. Labs were significant for elevated c-reactive protein and leukocytosis. CT abdomen revealed segmental wall thickening of the descending colon with adjacent inflammation and associated with diverticular disease favoring acute diverticulitis (Figs 1 and 2). There was no evidence of nephrolithiasis. The patient was discharged on a 7-day course of Augmentin and was scheduled for colonoscopy.

**Figure 3:**
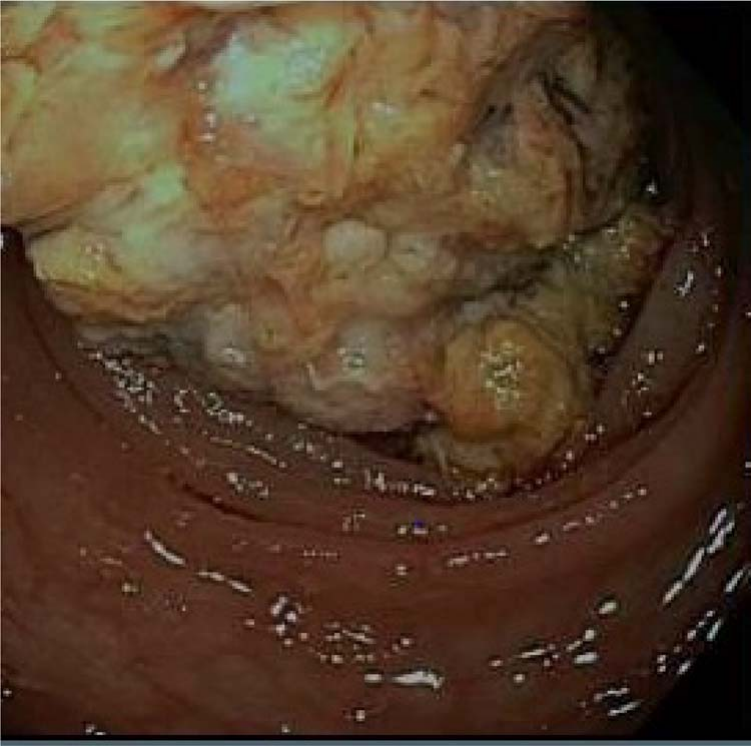
A fungating partially obstructing mass was found in the descending colon on colonoscopy.

After discharge, the patient continued to experience intermittent abdominal pain, melena and diarrhea. These symptoms prompted three more emergency room visits over a 1-month period. Repeat abdominal imaging (CT scan) revealed previously noted colonic inflammation. In addition, colonic intussusception was noted this time. The patient underwent a colonoscopy 2 weeks later revealing a fungating partially obstructing descending colon mass acting as the lead point for intussusception (Fig. 3). The mass was deemed too large for endoscopic removal and multiple biopsies were obtained. These biopsies revealed necrotic granulation tissue without any evidence of malignancy. Patient subsequently underwent an uneventful laparoscopic left hemicolectomy and was discharged home on postoperative Day 3. Final pathology revealed moderately differentiated adenocarcinoma, invading into pericolonic adipose tissue and metastases in 3 of 48 lymph nodes. Immunohistochemistry analysis revealed absence of MLH1, PMS2 and MSH and presence of PSH2, concerning for Lynch Syndrome. Further testing of MLH1 promoter was negative for hypermethylation. The patient was referred to medical oncology for adjuvant chemotherapy. She remains disease free and is on active surveillance for colorectal cancer in setting of Lynch Syndrome.

## DISCUSSION

The clinical presentation of adult intussusception varies considerably, with the most common symptoms including abdominal pain, nausea, vomiting, change in bowel habits, gastrointestinal bleeding and abdominal distention. The presentation of symptoms is consistent with the obstructive nature of intussusception. Typically, the onset and duration of symptoms in large bowel intussusception are significantly longer than in small bowel obstruction, which was true for our patient in this case report who had had a year of vague symptoms before first presenting to the ED. It is important to recognize that the presentation of intussusception has significant overlap with that of adenocarcinoma of the colon due to the obstructive nature of both.

Plain abdominal films, ultrasound and CT are all imaging modalities that can be used to evaluate a suspected case of intussusception. Plain films typically reveal signs of intestinal obstruction or perforation and can include distended loops of bowel with absence of gas [3]. Abdominal ultrasonography is an appropriate initial diagnostic technique in both children and adults; the sensitivity and specificity of ultrasound in diagnosing intussusception approach nearly 100% in experienced hands [4]. Classic features of intussusception seen with ultrasound include the target and doughnut signs on transverse view and pseudokidney on longitudinal view [5, 6]. CT is currently the most sensitive imaging method for detecting intussusception, with characteristic features including edematous bowel wall and mesentery within the lumen of the bowel wall [7]. Despite the consistent nature of CT in diagnosing intussusception, it is not always reliable at distinguishing a neoplastic lead point from a thickened bowel wall, as demonstrated in this case and in several others [8]. Colonoscopy is an appropriate tool for evaluation of intussusception, especially when there is concern for large bowel obstruction because it allows for the lesion to be diagnosed and biopsied. Although colonic intussusception is rare finding in general, descending colonic intussusception of the descending colon is even more uncommon because of its fixation to the retroperitoneum, with only a few cases reported in the literature [9]. Colonic intussusception is more likely to occur in the mobile portions of the colon such as in the cecum or sigmoid colon.

Treatment of intussusception varies between adults and children. In pediatric cases, it is appropriate to attempt to reduce the intussusception using barium enema. In adults, however, this approach is not advised due to the concern for malignancy [9]. Surgery is the primary management in adults with intussusception produced by an underlying lead point.

## CONCLUSION

Intussusception in adults is often the result of a pathologic lead point, with benign or malignant neoplasm accounting for two-thirds of cases. Of these neoplasms, 50% are malignant. The majority of cases of adult intussusception arise within the small bowel. Of those that do arise within the large bowel, the descending colon is an uncommon location due to its retroperitoneal attachments. Appropriate management of cases of adult intussusception includes imaging to aid in diagnosis followed by colonoscopy and surgical resection in cases where surgery is indicated.
